# Predictive risk modelling of high resource users under different prescription drug coverage policies in Ontario and Manitoba, Canada

**DOI:** 10.1186/s12913-023-09722-y

**Published:** 2023-07-19

**Authors:** Kathy Kornas, Joykrishna Sarkar, Randall Fransoo, Laura C. Rosella

**Affiliations:** 1 Dalla Lana School of Public Health, University of Toronto, Health Sciences Building 6th floor, 155 College Street, Toronto, ON M5T 3M7 Canada; 2 Manitoba Centre for Health Policy, University of Manitoba, Winnipeg, MB Canada; 3 ICES, 2075 Bayview Avenue, Toronto, ON M4N 3M5 Canada; 4 Institute for Better Health, Trillium Health Partners, 100 Queensway West, Mississauga, ON L5B 1B8 Canada; 5 Laboratory Medicine and Pathobiology, Temerty Faculty of Medicine, Simcoe Hall, 1 King’s College Cir, Toronto, ON M5S 1A8 Canada

**Keywords:** Health care utilization, High resource users, Prescription drug coverage

## Abstract

**Introduction:**

Studying high resource users (HRUs) across jurisdictions is a challenge due to variation in data availability and health services coverage. In Canada, coverage for pharmaceuticals varies across provinces under a mix of public and private plans, which has implications for ascertaining HRUs. We examined sociodemographic and behavioural predictors of HRUs in the presence of different prescription drug coverages in the provinces of Manitoba and Ontario.

**Methods:**

Linked Canadian Community Health Surveys were used to create two cohorts of respondents from Ontario (n = 58,617, cycles 2005–2008) and Manitoba (n = 10,504, cycles 2007–2010). HRUs (top 5%) were identified by calculating health care utilization 5 years following interview date and computing all costs in the linked administrative databases, with three approaches used to include drug costs: (1) costs paid for by the provincial payer under age-based coverage; (2) costs paid for by the provincial payer under income-based coverage; (3) total costs regardless of the payer (publicly insured, privately insured, and out-of-pocket). Logistic regression estimated the association between sociodemographic, health, and behavioral predictors on HRU risk.

**Results:**

The strength of the association between age (≥ 80 vs. <30) and becoming an HRU were attenuated with the inclusion of broader drug data (age based: OR 37.29, CI: 30.08–46.24; income based: OR 27.34, CI: 18.53–40.33; all drug payees: OR 29.08, CI: 19.64–43.08). With broader drug coverage, the association between heavy smokers vs. non-smokers on odds of becoming an HRU strengthened (age based: OR 1.58, CI: 1.32–1.90; income based: OR 2.97, CI: 2.18–4.05; all drug payees: OR 3.12, CI: 2.29–4.25). Across the different drug coverage policies, there was persistence in higher odds of becoming an HRU in low income households vs. high income households and in those with a reported chronic condition vs. no chronic conditions.

**Conclusions:**

The study illustrates that jurisdictional differences in how HRUs are ascertained based on drug coverage policies can influence the relative importance of some behavioural risk factors on HRU status, but most observed associations with health and sociodemographic risk factors were persistent, demonstrating that predictive risk modelling of HRUs can occur effectively across jurisdictions, even with some differences in public drug coverage policies.

**Supplementary Information:**

The online version contains supplementary material available at 10.1186/s12913-023-09722-y.

## Introduction

Health care spending around the world is concentrated among a small proportion of the population, known as high resource users (HRUs) [1]. In a review of studies of high-cost patients, including from Canada and the USA, the top 5% of health care users accounted for about 55% of total health care costs within a given year [2]. Pharmaceuticals are an important contributor to the overall healthcare costs of HRUs [2]. This is partly because HRUs, and those on the path of becoming an HRU, typically have chronic conditions for which medications are prescribed to treat and manage [3].

Studying HRUs across jurisdictions is a challenge due to variation in data availability and health services coverage. Costing methodologies for estimating healthcare costs and classifying patients as HRUs are commonly applied from the perspective of the health system provider, where only the costs incurred by the public healthcare payer are considered [4, 5]. In the Canadian context, the provincial differences in public drug plans results in variations in how costs from the pharmaceutical sector are included to identify HRUs in the population. In Canada, all provinces provide comparable universal health care coverage for hospital and physician services, but public insurance for out-of-hospital prescription medications are not covered by a national plan [6]. Eligibility for public drug plans differs across the provinces, depending on age, income, and other factors [7]. In 2018, only 42% of total prescription drug expenditures in Canada were covered under a provincial drug plan, with the remainder of drug costs financed through private insurance plans (37%) or individuals paying out-of-pocket (21%) [8]. There are also provincial differences in the availability of comprehensive drug data; for some provinces, dispensed prescriptions covered by private insurance or paid out-of-pocket are either not available in administrative data holdings or are limited to certain classes of drugs (e.g., Narcotics Monitoring System in Ontario) [9].

Understanding the risk factors associated with HRUs can inform efforts to curb growing health care costs. Previous studies have identified patient characteristics associated with becoming an HRU, including higher age, the presence of multiple chronic conditions, lifestyle behaviours, such as smoking and physical inactivity, and socioeconomic factors such as low income [3, 10–13]. However, in the context of health services research, differences in administrative data availability can have implications for identifying and predicting HRUs, for example, if drug data information is excluded for a portion of the population, whether because of data gaps or the use of the public health payer perspective. An important consideration is whether the predictors of HRUs persist with the inclusion of incomplete versus complete drug costs.

The different drug plans available in the provinces of Manitoba and Ontario, and complex data linkages available within the Manitoba Centre for Health Policy and Ontario’s ICES offers a unique opportunity to explore the predictors of HRUs under different access to drug administrative data. The objective of this study was to examine sociodemographic and behavioural predictors of HRUs in the presence of different prescription drug coverages in the two provinces and with the availability of broader drug data used to identify HRUs.

## Methods

### Context and setting

The study was conducted using population based data from the provinces of Ontario and Manitoba, Canada. Within each province, all residents receive universal health coverage from their single payer health insurance system: the Ontario Ministry of Health and Long Term Care and the Manitoba Ministry of Health, Seniors, and Active Living. During the study period, Ontario offered an age-based public drug-plan (i.e., Ontario Drug Plan) which covered drug costs for seniors aged 65 and over, whereas Manitoba offered an income-based plan (i.e., Pharmacare), in which drug costs for all family members were covered after a deductible was reached based on the total adjusted family income [7]. In both provinces, social assistance recipients received coverage under the public drug plans [7]. Health administrative data on utilization of publicly funded health care services across health sectors, as well as population survey data, are housed at ICES in Ontario and in the Manitoba Centre for Health Policy. The study was approved by the Research Ethics Boards at the University of Toronto, Sunnybrook Health Sciences Centre, and University of Manitoba.

### Data sources

Two study cohorts were created by linking respondents from the Canadian Community Health Surveys (CCHS) to provincial health administrative data. CCHS cycles in which important HRU risk factors were asked consistently in both provinces were combined using the pooled approach to maximize sample size [14]. The Ontario cohort consisted of respondents from the CCHS cycles 3.1 (2005) and 07/08, and the Manitoba cohort was created with CCHS cycles 07/08 and 09/10. The CCHS is a cross-sectional survey administered by Statistics Canada that collects self-reported health-related data and uses a probability sample and weighting system that is representative of 98% of the Canadian population aged 12 years and older living in private dwellings. Excluded from the CCHS sampling frame are individuals living in First Nation communities and other Aboriginal settlements, full-time members of the Canadian Forces, individuals living in long-term care institutions, and residents of certain remote regions. The detailed survey methodology of the CCHS is described elsewhere [15].

Data on health services utilized after CCHS interview date were obtained from the linked health administrative databases. Specific data sources used to compute health care costs for health services utilized across sectors is described in Table 1. A description of these health administrative databases is available in the Appendix, Supplement 1. In Ontario, drug data availability was limited to pharmaceuticals paid for by the provincial health system, which captured prescriptions for individuals aged 65 and older and those receiving social assistance. In Manitoba, drug data was available for the total cohort, including pharmaceuticals paid for by the province’s public drug plan and prescriptions paid by individuals out-of-pocket or by third-party insurance.

**Table 1:** Administrative databases used to compute health care costs for health care services utilized across sectors

Health Care Service	Data Source	
	Manitoba Cohort	Ontario Cohort
**Pharmaceuticals**	Drug Program Information Network	Ontario Drug Program
**Physician services**	Medical Services Data	Ontario Health Insurance Plan
**Inpatient hospital**	Discharge Abstract Database	Discharge Abstract Database
**Rehabilitation**	National Rehabilitation Reporting System	National Rehabilitation Reporting System
**Long-term care**	Long Term Care Utilization	Continuing Care Reporting System
**Complex continuing care**	Not available	Continuing Care Reporting System
**Same day surgery**	Discharge Abstract Database; Case Mix Grouper data	National Ambulatory Care Reporting System Metadata
**Emergency department**	Not available	National Ambulatory Care Reporting System Metadata
**Home care**	Not available	Ontario Home Care Administrative System; Home Care database
**Inpatient mental health**	Not available	Ontario Mental Health Reporting System
**Medical devices**	Not available	Assisted Devices Program

### Study population

The study population included CCHS respondents from Ontario and Manitoba, aged 18 years and older, who had a valid health card and agreed to have their survey responses linked to their provincial health administrative data. For individuals that appeared in multiple CCHS cycles, only data collected from their first CCHS interview was used. After exclusions, the Ontario cohort consisted of 58,617 CCHS respondents and the Manitoba cohort consisted of 10,504 CCHS respondents.

### HRU status ascertainment

Annual health care costs for five years following the respondent’s CCHS interview date were computed by applying a person-centered costing algorithm to the linked health administrative databases [5]. The costing algorithm calculated individual-level costs based on the utilization of health care services, including physician services, inpatient hospital stay, inpatient rehabilitation, long-term care, stays in complex continuing care hospitals, same day surgery, emergency department visits, home care, inpatient psychiatric admissions, medical devices, and prescriptions; the costing methodology is described elsewhere [5]. Given differences in drug coverage and data availability in the provinces, three different approaches were used to include drug costs in the costing algorithm.

#### Approach 1: total health system costs including drugs paid for by the provincial payer under age-based coverage

Based on the Ontario health system’s age-based drug plan, total health system costs included provincial payer funded prescriptions dispensed to seniors aged 65 and over and those receiving social assistance, along with other health sector costs that were estimated from the healthcare payer perspective. This approach was applied to the Ontario and Manitoba cohort.

#### Approach 2: total health system costs including drugs paid for by the provincial payer under income based coverage

Based on the Manitoba health system’s income-based drug plan, total health system costs included provincial payer funded prescriptions dispensed to the general population aged 18 and over, along with other health sector costs that were estimated from the healthcare payer perspective. This approach was applied to the Manitoba cohort.

#### Approach 3: total health system costs including all drug costs, regardless of payer

Leveraging all available drug data in Manitoba, total health system costs included provincial payer funded prescriptions as well as drugs paid for out-of-pocket and by third party insurance, along with other health sector costs that were estimated from the healthcare payer perspective. This approach was applied to the Manitoba cohort.

For each approach, individual per-person health care costs were ranked in each year and cost gradients were categorized as the top 5% and bottom 95%. HRUs were defined as the top 5% of health care users in any given year.

### HRU risk factors

HRU predictor variables were obtained from the CCHS and drawn from a Canadian validated population-based risk algorithm of adults who were likely to become the top 5% of health care users over the subsequent five years [13].

#### Sociodemographic variables

Sociodemographic risk factors included sex, age category (< 30, 30–39, 40–49, 50–59, 60–69, 70–79, ≥ 80), ethnicity (white, non-white), immigrant status (Canadian-born, immigrant less than 10 years, immigrant 10 or more years), household income quintile, and household food security (moderately/severely food insecure, food secure).

#### Health status variables

Health status risk factors included chronic conditions (self-reported having any of the following: asthma, arthritis, back problems, migraines, chronic obstructive pulmonary disease, diabetes, hypertension, heart disease, cancer, intestinal ulcers, stroke, urinary incontinence, bowel disease, Alzheimer’s, mood disorder, or anxiety) and self-reported general health (excellent/very good/good, fair, poor).

### Body Mass Index and Health behavioural variables

Body mass index (BMI) was calculated by dividing self-reported body weight by the square of body height (kg/m^2^) and classified according to the international standard [16]: underweight (< 18.5 kg/m^2^); normal weight (18.5–24.9 kg/m^2^); overweight (25.0-29.9 kg/m^2^); obesity class I (30.0-34.9 kg/m^2^); obesity class II (35.0-39.9 kg/m^2^); and obesity class III (≥ 40.0 kg/m^2^). Health behavioural risk factors included smoking, physical inactivity, and alcohol consumption. Smoking status was categorized as current heavy smoker (≥ 1 pack (25 cigarettes)/day); current light smoker (< 1 pack (25 cigarettes)/day), former heavy smoker (≥ 1 pack (25 cigarettes)/day); former light smoker (< 1 pack (25 cigarettes)/day); and non-smoker (never smoker or former occasional smoker with < 100 lifetime cigarettes). Physical activity was measured by the average daily energy expended for leisure time activities, calculated by multiplying the number of times engaged in each type of activity in the past year, average duration of participation in hours, and metabolic equivalent of task (MET) value assigned to each activity [17]. Respondents were categorized as being inactive (0-1.4 kcal/kg/day); moderately inactive (1.5-3.0 kcal/kg/day), and active (≥ 3.0 kcal/kg/day). Physical activity was further categorized into physical activity quartiles ranging from 1 (the 25% that were least physically active) to 4 (the 25% that were most physically active). Alcohol consumption was categorized according to sex-specific cut-offs for the number of alcoholic drinks consumed in the previous week: heavy drinker (≥ 21 (men) or ≥ 14 (women) drinks, or binging behaviour on a weekly basis (≥ 5 drinks on any occasion)); moderate drinker (4–21 (men) or 3–14 (women) drinks); light drinker (1–3 (men) or 1–2 (women) drinks); and non-drinker (did not consume alcohol in the last 12 months or drinks less than weekly). The CCHS questions used to define the risk factor variables are reported elsewhere [13].

### Statistical analysis

Using the sample survey weights, weighted estimates (frequencies) were calculated to determine the distribution of sociodemographic, health status and health behavioural characteristics of the study cohorts. Characteristics were reported under the three different approaches used to define HRUs.

Logistic regression models were used to estimate adjusted odds ratios and 95% confidence intervals (CIs) for the association between risk factor variables and becoming an HRU in the five years following CCHS interview. Missing values for risk factors were included as separate categories, except for chronic conditions, for which respondents with missing information were dropped. Model 1 was estimated with the outcome of HRU status defined using approach 1 (including costs from provincially funded age-based drug coverage); model 2 defined HRUs using approach 2 (including costs from provincially funded income-based drug coverage); and model 3 defined HRUs using approach 3 (including all drug costs regardless of payer). All models included the same predictor variables.

All analyses were weighted using sampling weights provided by Statistics Canada to adjust for the complex sampling design of the CCHS and produce estimates representative of the provincial population. We rescaled the survey weights to account for pooled CCHS cycles by dividing the survey weights by the number of cycles combined (i.e., the sum of the weights scaled by 2 in the Ontario and Manitoba cohorts). This approach represents the average population that covers the combined time periods of the individual CCHS cycles [14]. CIs were estimated using bootstrap weights applied using the balanced repeated replication approach for standard error estimation. All statistical analyses were conducted using SAS, version 9.4 (SAS institute, Cary, NC).

## Results

Weighted baseline characteristics for the Ontario and Manitoba cohorts are presented in Table 2. The sex and age distribution was similar in both the Ontario and Manitoba cohorts. The Ontario cohort had a larger proportion of immigrants, people with non-white ethnicity, and people in the highest income quintile than the Manitoba cohort. The proportion reporting the presence of at least one chronic condition was similar in both cohorts, as was the distribution of behavioural characteristics.

**Table 2:** Weighted^a^ population distribution of characteristics in the Ontario and Manitoba cohorts

Risk Factor	Ontario Cohort Overall	Manitoba Cohort Overall
Unweighted Weighted population	n = 58,617 9,368,436	n = 10,504 1,475,359
**Sex** (Male)	48.9	49.3
**Age group**		
<30	21.6	21.4
30–39	18.6	17.1
40–49	22.1	18.9
50–59	16.9	17.7
60–69	11.3	13.0
70–79	6.7	7.5
≥80	2.9	4.4
**Ethnicity**		
White	76.2	79.8
Non-white	21.2	9.6
**Immigrant status**		
Canadian-born	67.2	84.3
Immigrant (< 10 years)	8.4	4.2
Immigrant (≥ 10 years)	24.2	11.3
**Household income**		
Q1 (lowest)	15.6	18.2
Q2	15.9	19.8
Q3	16.9	19.2
Q4	18.9	17.6
Q5 (highest)	20.0	14.2
**Food Security**		
Food Secure	93.1	92.7
Food Insecure	5.3	5.8
**Chronic Condition**		
Yes	56.3	58.6
No	43.4	35.7
**General Health**		
Excellent/very/good	89.1	87.4
Fair	8.1	10.0
Poor	2.8	2.6
**Body Mass Index**		
Underweight	2.6	2.0
Normal weight	44.4	37.9
Overweight	33.2	34.4
Obese class I	11.3	13.6
Obese class II	3.0	4.6
Obese class III	1.4	2.1
**Smoking Status**		
Heavy smoker	3.5	3.5
Light smoker	17.8	18.6
Former heavy smoker	6.2	7.5
Former light smoker	16.1	16.7
Non-smoker	52.9	50.1
**Physical activity**		
Inactive	49.2	48.4
Moderately active	24.3	24.7
Active	24.4	25.0
**Alcohol consumption** ^**b**^		
Heavy drinker	8.4	7.8
Moderate drinker	21.6	18.6
Light drinker	13.8	14.6
Non-drinker	54.8	58.4

^**a**^Numbers are weighted percentages using bootstrap weights provided by Statistics Canada. Column percentages do not total 100% where missing values are not reported

^b^ Non-drinker defined as those who did not consume alcohol in the last 12 months or drinks less than weekly

Table 3 summarizes the extent to which adjusted odds ratios for 5-year HRU risk changed when different approaches were used to include drug data in the calculation of health system costs.

**Table 3:** Weighted ^a^ adjusted odds ratios of the multinomial logistic models predicting 5-year HRU status in Ontario and Manitoba under different access to drug coverage

	Ontario Cohort	Manitoba Cohort		
Inclusion of drug data in total health system costs ^b^	Approach 1 age-based coverage	Approach 1 age-based coverage	Approach 2 income-based coverage	Approach 3 drug costs from all payers
**Risk Factor**	OR^a^ (95% CI)	OR^a^ (95% CI)	OR^a^ (95% CI)	OR^a^ (95% CI)
**SOCIODEMOGRAPHICS**				
**Sex** (Male vs. Female)	**1.27** (1.18–1.38)	**1.28** (1.10–1.49)	**1.42** (1.22–1.66)	**1.42** (1.22–1.66)
**Age group**				
18–30	**reference**	**reference**	**reference**	**reference**
30–39	1.13 (0.88–1.44)	1.03 (0.63–1.67)	1.29 (0.83-2.00)	1.38 (0.89–2.14)
40–49	**2.51** (2.05–3.08)	**2.99** (2.03–4.39)	**2.50** (1.72–3.64)	**2.67** (1.83–3.88)
50–59	**5.11** (4.19–6.23)	**4.84** (3.32–7.05)	**3.82** (2.65–5.49)	**3.94** (2.73–5.68)
60–69	**10.55** (8.66–12.85)	**7.27** (4.98–10.61)	**6.12** (4.25–8.82)	**6.13** (4.24–8.87)
70–79	**16.58** (13.54–20.31)	**12.92** (8.78–19.01)	**8.94 (**6.13–13.03)	**9.43** (6.45–13.79)
≥80	**37.29** (30.08–46.24)	**36.73** (24.59–54.84)	**27.34** (18.53–40.33)	**29.08** (19.64–43.08)
**Ethnicity**				
White	**reference**	**reference**	**reference**	**reference**
Non-white	**0.78** (0.69–0.89)	1.18 (0.84–1.65)	1.12 (0.79–1.58)	1.26 (0.89–1.76)
Missing	1.01 (0.79–1.28)	0.97 (0.75–1.25)	**1.57** (1.14–2.16)	0.91 (0.70–1.17)
**Immigrant status**				
Canadian-born	**reference**	**reference**	**reference**	**reference**
Immigrant (< 10 years)	**0.79** (0.63-1.00)	**0.14** (0.05–0.39)	**0.09** (0.03–0.31)	**0.08** (0.23–0.29)
Immigrant (≥ 10 years)	0.96 (0.88–1.05)	1.00 (0.79–1.26)	1.05 (0.83–1.32)	1.00 (0.79–1.26)
Missing	1.19 (0.60–2.40)	0.54 (0.14–2.13)	0.60 (0.15–2.39)	0.61 (0.15–2.44)
**Household income**				
Q1 (lowest)	**1.69** (1.47–1.95)	**1.35** (1.01–1.79)	**1.40** (1.04–1.88)	1.20 (0.90–1.60)
Q2	**1.50** (1.30–1.72)	**1.50** (1.13–1.98)	**1.53** (1.14–2.03)	**1.33** (1.01–1.76)
Q3	**1.22** (1.06–1.41)	1.06 (0.79–1.41)	1.11 (0.82–1.49)	1.02 (0.77–1.36)
Q4	**1.25** (1.09–1.44)	1.02 (0.76–1.39)	1.08 (0.79–1.47)	0.99 (0.77–1.36)
Q5 (highest)	**reference**	**reference**	**reference**	**reference**
Missing	**1.47** (1.27–1.70)	1.17 (0.85–1.61)	**1.57** (1.14–2.16)	1.35 (0.98–1.84)
**Food Security**				
Food Secure	**0.77** (0.65–0.90)	1.26 (0.92–1.74)	**1.42** (1.02–1.97)	**1.45** (1.04–2.01)
Food Insecure	**reference**	**reference**	**reference**	**reference**
Missing	**0.45** (0.30–0.68)	**1.98** (1.12–3.51)	**1.84** (1.03–3.28)	1.78 (0.99–3.19)
**HEALTH STATUS**				
**Chronic Condition**				
Yes vs. No	**1.44** (1.30–1.59)	**1.68** (1.38–2.05)	**1.69** (1.39–2.07)	**1.70** (1.39–2.07)
**General Health**				
Excellent/very/good	**reference**	**reference**	**reference**	**reference**
Fair	**1.50** (1.36–1.67)	**2.45** (1.99–3.01)	**2.48** (2.02–3.06)	**2.57** (2.09–3.16)
Poor	**2.89** (2.52–3.32)	**3.54** (2.64–4.76)	**3.83** (2.85–5.14)	**3.84** (2.86–5.16)
Missing	**1.16** (0.33–4.05)	0.85 (0.09–7.65)	0.19 (0.01–6.24)	0.21 (0.01–6.47)
**BMI and HEALTH BEHAVIOURS**				
**Body Mass Index**				
Underweight	1.02 (0.77–1.35)	**1.87** (1.12–3.12)	**1.90** (1.14–3.18)	**1.88** (1.12–3.16)
Normal weight	**reference**	**reference**	**reference**	**reference**
Overweight	1.08 (0.99–1.18)	1.09 (0.92–1.30)	1.09 (0.92–1.31)	1.14 (0.96–1.36)
Obese class I	1.12 (0.99–1.26)	1.02 (0.82–1.27)	1.08 (0.87–1.34)	1.16 (0.93–1.44)
Obese class II	**1.44** (1.19–1.74)	0.91 (0.63–1.30)	0.95 (0.66–1.35)	0.97 (0.67–1.39)
Obese class III	**1.89** (1.46–2.45)	1.28 (0.82-2.00)	**1.67** (1.08–2.55)	**1.89** (1.25–2.87)
Missing	1.10 (0.89–1.36)	1.18 (0.76–1.83)	1.03 (0.65–1.63)	1.07 (0.68–1.69)
**Smoking Status**				
Heavy smoker	**1.58** (1.32–1.90)	**2.29** (1.65–3.18)	**2.97** (2.18–4.05)	**3.12** (2.29–4.25)
Light smoker	**1.29** (1.15–1.45)	**1.84** (1.48–2.28)	**1.75** (1.40–2.18)	**1.86** (1.49–2.30)
Former heavy smoker	**1.34** (1.18–1.52)	**1.51** (1.19–1.92)	**1.52** (1.19–1.93)	**1.55** (1.21–1.97)
Former light smoker	**1.14** (1.03–1.26)	**1.34** (1.10–1.63)	**1.48** (1.21–1.79)	**1.49** (1.23–1.81)
Non-smoker	**reference**	**reference**	**reference**	**reference**
Missing	0.94 (0.77–1.13)	1.00 (0.70–1.42)	0.92 (0.64–1.33)	0.95 (0.66–1.37)
**Physical activity Quartile**				
Q1 (lowest)	**1.19** (1.06–1.34)	1.06 (0.85–1.33)	**1.28** (1.02–1.61)	1.22 (0.97–1.54)
Q2	0.99 (0.88–1.11)	1.13 (0.90–1.42)	1.26 (0.99–1.59)	**1.31** (1.04–1.64)
Q3	1.02 (0.90–1.15)	1.04 (0.83–1.31)	1.17 (0.93–1.48)	1.16 (0.92–1.47)
Q4 (highest)	**reference**	**reference**	**reference**	**reference**
Missing	**1.42** (1.13–1.78)	1.03 (0.57–1.84)	1.35 (0.74–2.46)	1.31 (0.72–2.38)
**Alcohol consumption**				
Heavy drinker	0.99 (0.82–1.19)	1.06 (0.74–1.52)	1.03 (0.72–1.48)	0.97 (0.68–1.39)
Moderate drinker	0.93 (0.81–1.06)	**0.63** (0.45–0.83)	0.69 (0.52–0.91)	**0.64** (0.48–0.84)
Light drinker	**reference**	**reference**	**reference**	**reference**
Non-drinker	1.04 (0.93–1.17)	1.19 (0.97–1.47)	1.22 (0.99–1.52)	1.21 (0.97–1.49)
Missing	1.06 (0.80–1.41)	**0.32** (0.11–0.97)	0.36 (0.12–1.08)	0.35 (0.12–1.05)

^**a**^OR = weighted odds ratio calculated using multinomial logistic regression. Bolded values indicate significantly different from reference category (p < 0.05)

^b^**Approach 1**: Health systems costs include drug costs paid for by the provincial payer under age-based coverage (seniors aged 65 + and those receiving social assistance)

**Approach 2**: Health system costs include drug costs paid for by the provincial payer under income-based coverage (adults aged 18+)

**Approach 3**: Health system costs include total drug costs, regardless of the payer

### Associations with sociodemographic risk factors

Using drug costs based on age-based coverage (approach 1), associations with sex and age on HRU risk were similar in the Ontario and Manitoba cohort, with higher odds observed for males and older age groups. Using drug costs based on income-based coverage (approach 2) and drug costs from all payers (approach 3), the association between males versus females on the odds of becoming an HRU increased, while the strength of the association between older age groups versus the youngest age group was attenuated.

Significant risk reductions were observed for those with non-white ethnicity (OR = 0.78, CI: 0.69–0.89) in the Ontario cohort only, with the use of drug costs from age-based coverage. In the models using age-based drug data in both provinces, being a recent immigrant (< 10 years) was associated with reduced HRU risk by 21% in the Ontario cohort (CI: 0.63-1.00) and 86% in the Manitoba cohort (CI: 0.05–0.39). The association observed for recent immigrants was strengthened when including drug costs based on income-based coverage (OR: 0.09, CI: 0.03–0.31) and drug costs from all payers (OR: 0.08, CI: 0.23–0.29).

All drug coverage approaches showed persistence in higher odds of becoming an HRU among residents in low income households compared to the highest income households, with no meaningful differences in the strength of the association. In the Ontario cohort, with access to drug costs from age-based coverage, food security was associated with 23% lower odds (CI: 0.65–0.90) of becoming an HRU compared to food insecurity. Converse trends were observed in the Manitoba cohort, with no significant association under access to drug costs from age-based coverage, 42% higher odds under access to drug costs from income-based coverage (CI: 1.02–1.97), and 45% higher odds under access to drug costs from all payers (CI: 1.04–2.01).

### Associations with health status factors

Across the different drug coverage approaches, no meaningful differences were observed in the association between reported chronic condition and odds of becoming an HRU. With drug costs from age-based coverage, poor perceived health was associated with higher odds of HRU in the Ontario cohort (OR: 2.89, CI: 2.52–3.32) and in the Manitoba cohort (OR: 3.54, CI: 2.64–4.76). The odds associated with poor perceived health strengthened using income-based drug costs (OR: 3.83, CI: 2.85–5.14) and drug costs from all payers (OR: 3.84, CI: 2.86–5.16).

### Associations with BMI and health behaviours

Using age-based drug coverage costs, obesity was associated with significant increases in the odds of HRU risk in the Ontario cohort, but not in the Manitoba cohort. However, significant associations were detected for obesity class I with access to income-based drug coverage (OR: 1.67, CI: 1.08–2.55) and using drug costs from all payers (OR: 1.89, CI: 1.25–2.87). Similarly, the observed associations between smoking status and odds of becoming an HRU strengthened using drug costs from all payers. In the Manitoba cohort, the largest increases in the association were observed among heavy smokers, for whom the odds of HRU were 2.29 under aged-based drug coverage (CI: 1.65–3.18), 2.97 under income-based drug coverage (CI: 2.18–4.05), and 3.12 under access to drug costs from all payers (CI: 2.29–4.25). No meaningful differences were observed across drug access policies and the associations between physical activity and alcohol consumption on odds of becoming an HRU.

## Discussion

In research and predictive modelling focused on HRUs of the health system, it is often necessary to define HRUs in a population of interest based on the available health administrative data and a costing perspective (e.g., health system provider, societal, or patient) [18]. In the HRU context, a consideration of using the health system provider costing perspective in modelling is that there are differences in drug coverage policies across jurisdictions, which results in differential access to drug data that are available, and inevitable differences in ascertaining HRU status. In addition, the ability to incorporate a patient costing perspective into HRU modelling relies on having access to data on health care costs, such as prescription drugs, incurred by patients. We used three different approaches to identify HRUs in the provinces of Ontario and Manitoba, based on the province’s drug coverage policies, available drug administrative data and comparable administrative data for other health services utilized under the provinces universal health system. In general, we found persistent associations of socioeconomic and health status risk factors on HRU status, namely household income and chronic conditions, regardless of differences in drug coverage policies and differences in how drug administrative data were included in defining HRUs. The strength of some demographic and health behavioural risk factor associations varied under different drug coverage policies. In particular, the association between age and HRU status attenuated under income-based coverage compared to age-based coverage, whereas associations with smoking were strengthened.

The differences in health behavioural risks observed across drug coverage approaches may be influenced by differential access to prescription medications for younger individuals (i.e., under the age of 65) who may not have access to medications to treat their health conditions, particularly under age-based coverage. One study estimated that 10% of Canadians have reported cost related non-adherence to prescription medications [19]. The development of multiple chronic conditions is a risk factor for both high health system resource use and use of multiple prescription medications [2, 20]. Many of the types of conditions that multimorbid patients are treated for are considered preventable [21]. For example, in Manitoba, the most prevalent conditions among those who were higher consumers of prescription drugs were largely preventable conditions such as hypertension, diabetes, ischemic heart disease, and depression [22]. The findings suggest that with age-based drug coverage and narrower inclusions of pharmaceutical data to identify HRUs in the population, the impact of risk factors relevant to prevention can be under-estimated.

Our findings of increased risk of becoming a HRU among non-drinkers compared to light drinkers were consistent across drug coverage approaches, and has been corroborated in previous research on high health care utilization [11, 23]. It is important to note that the CCHS measures alcohol consumption for the 12 months prior and does not provide information on past alcohol consumption behaviours over the person’s lifetime. It is reasonable that an individual’s health declines, medical contradictions and recommendations from health professionals would influence health behaviour change, such as abstaining from or reducing alcohol intake. Given data limitations, we were unable to explore the associations with HRUs and drinking patterns in more depth in the current study, which represents an area for future research.

The reduced HRU risk observed among recent immigrants was similar across drug approaches, with a stronger protective effect observed in Manitoba compared to Ontario. Immigrants in Canada have a health advantage, known as the healthy immigrant effect, in which they have been shown to have generally better health than their Canadian-born counterparts [24]. The provincial differences in the magnitude of the healthy immigrant effect for HRU status is likely because of immigration policies. Specifically, most of the new immigrants to Canada choose to settle in the major cities, namely Toronto (Ontario), Montreal (Quebec) and Vancouver (British Columbia), given labour force growth and existing networks with ethnocultural groups in these regions. To broaden the regional distribution of immigrants, Manitoba (and other provinces) have introduced programs with incentives to attract new immigrants who can contribute to the labour force, including the Manitoba Provincial Nominee Program [25]. Under this program, Manitoba saw an increase of well-educated young immigrants between 25 and 49 years old, which likely contributes to the observed healthy immigrant effect in Manitoba [25].

This analysis is strengthened by linkages of health administrative data with population survey data, which allowed for the creation of provincially-representative cohorts with information on socioeconomic and health behavioural information, which is often not available in administrative data alone. Few jurisdictions have access to similar data linkages. Despite these strengths, there are some notable limitations to our study. The CCHS sampling frame pertains to adults living in private dwellings, and therefore excludes institutionalized persons living in long term care, complex continuing care facilities, mental health institutions or hospitals. It is expected that some individuals that become a HRU reside in these facilities or experience transient living situations, such as homelessness, and would not be represented in our analyses. This would affect external generalizability to the broader Ontario and Manitoba population, but not internal validity since health care utilization was ranked within the CCHS population so relative cost categories are accurate within the study population. Given the CCHS sampling frame, the study findings should be interpreted as pertaining to the general household population, and not the total population, particularly residents of institutions or people experiencing homelessness. Further, the CCHS could only be linked to health administrative data for those who agreed to linkage and provided a valid health card number, which was 80% in the Ontario and Manitoba cohort. An evaluation of CCHS Cycle 1.1 linkage with health administrative data found similar coverage rates for sex and age groups except for seniors aged 75 years and over who had significantly lower coverage (70%) compared to the younger age group 12 and 74 years old (92%) [26]. Therefore, the linked CCHS cohort shows acceptable coverage, but the potential for selection bias should be considered when interpreting estimates, particularly for people aged 75 or older who have lower coverage rates. Our analyses did not include costs for over the counter medications that were incurred by patients, such as costs for smoking cessation products. Our study also did not examine geographic variables, which represents an area for future research given that previous research has shown geographic variation in HRU incidence among seniors [27]. In addition, the use of self-reported measures are subject to reporting biases, such as social desirability and recall bias. However, it is important to recognize that measuring health behaviours at the population level is difficult to achieve any other way. Lastly, in interpreting the findings, it is important to consider changes in population demographics and the prevalence of behavioural risk factors over time.

## Conclusion

When applying the health system provider perspective in the context of a universal health system, it is common to ascertain HRU status using health administrative data for publicly funded services. Our findings caution that the ascertainment of HRU status can vary with different drug coverage policies, resulting in somewhat different levels of risk associated with certain HRU predictors. In general, most of the health and sociodemographic risk factors examined in our study showed persistent associations with HRU status across drug coverage approaches, but some risk factors, particularly health behavioural risks, were more sensitive to situations where there is less comprehensive drug coverage. Overall, we demonstrate that HRU research can occur effectively across jurisdictions, despite difference in public drug coverage policies.

## Electronic supplementary material

Below is the link to the electronic supplementary material.

**Supplementary Material 1**: Description of Data Sources Used to Compute Health Care Costs in Manitoba and Ontario

## Data Availability

The dataset for this study is held securely in coded form at ICES and the Manitoba Centre for Health Policy (MCHP). While legal data sharing agreements prohibit ICES and MCHP from making the dataset publicly available, access may be granted to those who meet pre-specified criteria for confidential access available at www.ices.on.ca/DAS (email: das@ices.on.ca) and www.umanitoba.ca/manitoba-centre-for-health-policy/data-repository (email: mchp_access@cpe.umanitoba.ca). The full dataset creation plan and underlying analytic code are available from the authors (LR) upon request, understanding that the computer programs may rely upon coding templates or macros that are unique to ICES and MCHP and therefore either inaccessible or may require modification.

## Abbreviations

BMI: body mass index
CCHS: Canadian Community Health Surveys
CI: confidence interval
HRU: high resource user
MET: metabolic equivalent of task
OR: odds ratio
