# Supplementary material for: Predictive risk modelling of high resource users under different prescription drug coverage policies in Ontario and Manitoba, Canada
Source: BMC Health Serv Res. 2023 Jul 19;23:768. doi: 10.1186/s12913-023-09722-y (PMC10357670; doi:10.1186/s12913-023-09722-y)
Supplement: Supplementary file 1 — Supplementary Material 1: Description of Data Sources Used to Compute Health Care Costs in Manitoba and Ontario [file 12913_2023_9722_MOESM1_ESM.docx]

**Appendix - Supplement 1**

**Description of Data Sources Used to Compute Health Care Costs in Manitoba and Ontario**

**Drug Program Information Network (DPIN)** – contains prescription drug claims from DPIN, an electronic, on-line, point-of-sale prescription drug database that connects Manitoba Health and all pharmacies in Manitoba. The DPIN system generates complete drug profiles for each client including all transactions at the point of distribution. Information about pharmaceutical dispensations, prescriptions identified as potential drug utilization problems, non-adjudicated prescriptions, and ancillary programs and non-drug products are captured in real time for all Manitoba residents, regardless of insurance coverage or final payer.

**Ontario Drug Program –** contains information on prescription drug claims covered by and made to the Ontario Drug Benefit programme. The programme primarily covers drug expenses for Ontarians with valid OHIP card who are 65 years and older or residents of long-term care facilities, but also provides coverage to those receiving social assistance.

**Medical Services Data –** Manitoba database consisting of claims for physician visits in offices, hospitals and outpatient departments; fee-for-service components for tests such as lab and x-ray procedures performed in offices and hospitals; payments for on-call agreements (e.g. anaesthetists) that are not attributed to individual patients; as well as information about physician specialties, and shadow billings.

**Ontario Health Insurance Plan (OHIP) –** contains information on inpatient and outpatient services provided to Ontario residents for the province’s publicly funded health insurance system by fee-for-service health care practitioners (primarily physicians) and shadow billings for those paid through non-fee-for-service payment plans.

**Discharge Abstract Database (DAD)** – compiled by the Canadian Institute for Health Information and contains administrative, clinical (diagnoses and procedures/interventions), demographic, and administrative information for all admissions to acute care hospitals, rehab, chronic, and day surgery institutions across Canada.

**National Rehabilitation Reporting System (NRS)** – compiled by the Canadian Institute for Health Information and contains client data collected from participating adult inpatient rehabilitation facilities and programs across Canada.

**Long Term Care Utilization –** The LTC program, originally known as the Personal Care Home program, consists of records of chronic and rehabilitative services provided by long term care institutions in Manitoba. Long term care may be provided in hospitals, in chronic care beds, or in personal care homes. These records include information on admissions, separations, assessments, levels of care, and rate changes.

**Continuing Care Reporting System (CCRS) –** compiled by the Canadian Institute for Health Information and contains demographic, clinical, functional, and resource utilization information for individuals receiving facility-based continuing care (also known as extended, auxiliary, or complex chronic care) in Ontario hospitals and residential care providing 24 hour nursing services.

**Case Mix Grouper Data –** supports the Case Mix Group (CMG) component of the Hospital Abstracts data, containing variables related to CMG codes, diagnosis codes, discharge date and times, and intervention codes.

**National Ambulatory Care Reporting System Metadata (NACRS) –** compiled by the Canadian Institute for Health Information and contains clinical (diagnoses and procedures), demographic, and administrative information for all patient visits made to hospital- and community-based ambulatory centres (emergency department, day surgery units, hemodialysis units, and cancer care clinics) in Ontario.

**Ontario Home Care Administrative System –** contains information on individuals receiving home care in Ontario. Main data elements include admittance date to home care, diagnoses associated with the application for home care and end date of home care services.

**Home Care Database –** a clinic centric database that captures all services that are provided by or coordinated by community care access centres. The data elements include client, intake, assessment, admission & discharge, diagnosis and surgical procedure, and care delivery.

**Ontario Mental Health Reporting System –** compiled by the Canadian Institute for Health Information and contains clinical (diagnoses and procedures), demographic, and administrative information for all admissions to adult designated inpatient mental health beds. This includes beds in general hospitals, provincial psychiatric facilities, and speciality psychiatric facilities.

**Assisted Devices Program –** provides funding to Ontario residents who have long-term physical disabilities to provide access to personalized assistive devices appropriate for individual’s basic needs. The data elements include general information on select devices, renewal, insulin pumps & supplies, home oxygen, respiratory equipment & supplies, and ventilator equipment & supplies.
